# The diverse roles of miRNAs in HIV pathogenesis: Current understanding and future perspectives

**DOI:** 10.3389/fimmu.2022.1091543

**Published:** 2023-01-05

**Authors:** Farooq Rashid, Silvere D. Zaongo, Fangzhou Song, Yaokai Chen

**Affiliations:** ^1^ Department of Infectious Diseases, Chongqing Public Health Medical Center, Chongqing, China; ^2^ Basic Medicine College, Chongqing Medical University, Chongqing, China

**Keywords:** HIV-1, miRNA, mRNAs, HAART, therapeutic potential

## Abstract

Despite noteworthy progress made in the management and treatment of HIV/AIDS-related disease, including the introduction of the now almost ubiquitous HAART, there remains much to understand with respect to HIV infection. Although some roles that miRNAs play in some diseases have become more obvious of late, the roles of miRNAs in the context of HIV pathogenesis have not, as yet, been elucidated, and require further investigations. miRNAs can either be beneficial or harmful to the host, depending upon the genes they target. Some miRNAs target the 3′ UTR of viral mRNAs to accomplish restriction of viral infection. However, upon HIV-1 infection, there are several dysregulated host miRNAs which target their respective host factors to either facilitate or abrogate viral infection. In this review, we discuss the miRNAs which play roles in various aspects of viral pathogenesis. We describe in detail the various mechanisms thereby miRNAs either directly or indirectly regulate HIV-1 infection. Moreover, the predictive roles of miRNAs in various aspects of the HIV viral life cycle are also discussed. Contemporary antiretroviral therapeutic drugs have received much attention recently, due to their success in the treatment of HIV/AIDS; therefore, miRNA involvement in various aspects of antiretroviral therapeutics are also elaborated upon herein. The therapeutic potential of miRNAs are discussed, and we also propose herein that the therapeutic potential of one specific miRNA, miR-34a, warrants further exploration, as this miRNA is known to target three host proteins to promote HIV-1 pathogenesis. Finally, future perspectives and some controversy around the expression of miRNAs by HIV-1 are also discussed.

## 1 Introduction

MicroRNAs (miRNAs) refer to non-coding RNAs (ncRNAs) which are approximately 22 nucleotides in length, and usually bind to the 3′ untranslated region (3′ UTR) of their target messenger RNA (mRNAs), to either promote mRNA degradation or to repress translation (1). miRNA biogenesis and maturation is a stepwise process that starts in the nucleus and ends in cytoplasm. Most miRNA genes are transcribed by RNA polymerase II (Pol II) into long primary transcripts of miRNA called pri-miRNAs harboring a hairpin structure (2–4). The pri-miRNA are processed within the nucleus by Drosha, an RNAse III enzyme, into stem-loop intermediates called precursor-miRNA (pre-miRNAs) (2). These pre-miRNAs are then transported from the nucleus to the cytoplasm where they are further processed by the Dicer enzyme. Dicer converts them into their mature forms, called miRNAs, which are ultimately responsible for the phenomenon of RNA silencing (2, 5).

miRNAs are involved in the regulation of several human diseases including cancer, liver diseases, neurodegenerative diseases, and autoimmune diseases, and therefore affects many developmental pathways and cellular processes (1, 6–9). Furthermore, miRNAs are known to play regulatory roles in the pathogenesis of different viral diseases, including in human immunodeficiency virus (HIV)-related disease (10).

HIV is a retrovirus with a single-stranded RNA genome. After entering the host cell, single-stranded RNA is reverse transcribed into DNA, which then integrates into host DNA (11, 12). HIV DNA integration is the crucial step of the viral life cycle which renders the infection challenging to cure, as the infected cell remains infected for the life of the cell (12). Host enzymes and miRNAs are actively involved in all processes of the HIV life cycle, from attachment to the host cell until the production of viable HIV virions (13–16).

Past evidence has demonstrated that miRNAs are involved not only in HIV pathogenesis before treatment, but also in the immune recovery process during antiretroviral therapy (ART), and may serve as biomarkers and even as predictive markers for immune responses after initiation of ART (17–19). This review aims to summarize major advancements made in the field of miRNAs and HIV. This work presents details of the functions of cell-encoded and viral-encoded miRNAs, and their regulation during the pathophysiological processes of HIV-1. Furthermore, the controversy surrounding HIV’s ability to express miRNA and the therapeutic potential of miRNAs for HIV-1-related disease are discussed. Finally, a chapter introducing future perspectives in this specific area of scientific endeavor concludes our review.

## 2 Interplay between host miRNAs and HIV-1

Host miRNAs play important roles in regulating HIV-1 infection, and the biological crosstalk between HIV-1 and host miRNAs has been studied in the past (10, 20). miRNAs regulate HIV-1 infection either directly, by interfering with the HIV genome, or indirectly, by regulating host factors which regulate HIV-1 pathogenesis (14, 21, 22).

### 2.1 Host miRNAs that directly target HIV-1

**Figure 1:**
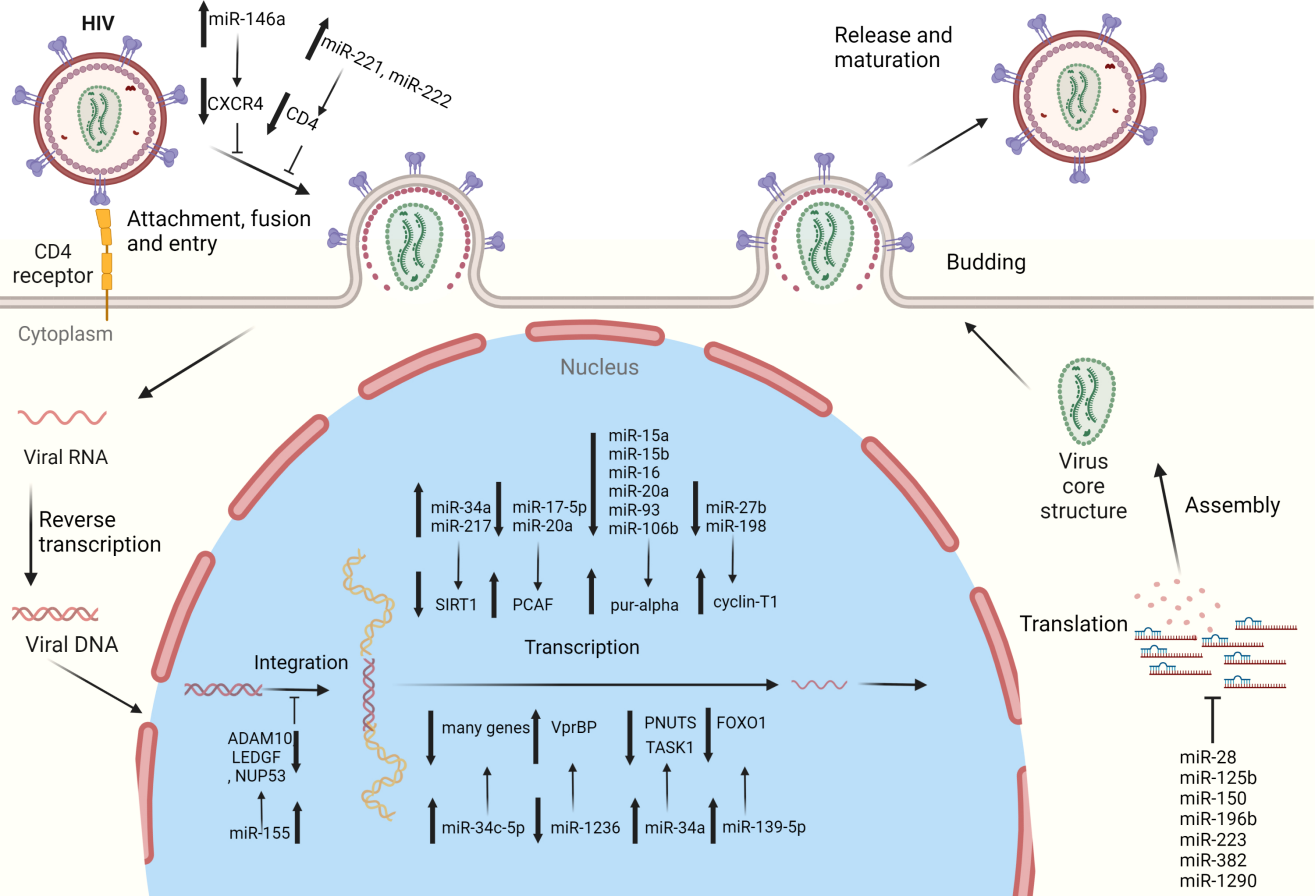
Cellular miRNAs regulating HIV-1 replication: several miRNAs have been identified which regulate HIV-1 replication and pathogenesis. These miRNAs target the specific host genes to regulate specific steps of the HIV-1 life cycle, i.e., cell entry, integration, replication, and latency.

The latent presence of HIV-1 virions in resting primary CD4^+^ T-cells is the major hurdle for the eradication of the HIV in patients on highly active antiretroviral therapy (HAART). The potential role that miRNAs may play in revealing the potential mechanism/s of this latency in resting CD4^+^ T-cells has been discussed previously (23). The 3′ UTR region of HIV-1 mRNAs contains binding sites for miR-28, miR-125b, miR-150, miR-223, and miR-382 **(** **Figure 1** **) (** **Table 1** **)**. Microarray analysis indicates that these miRNAs are highly abundant in resting CD4^+^ T-cells, compared to activated CD4^+^ T-cells. However, when resting CD4^+^ T-cells are activated, this results in the downregulation of all the above-mentioned miRNAs. Therefore, the higher expression level of these miRNAs in resting CD4^+^ T-cells may be seen to be responsible for maintaining HIV-1 latency (23). These miRNAs have been found to be enriched in monocytes as opposed to macrophages, because upon differentiation, macrophages suppress the expression of miRNAs, to promote HIV-1 infection (26). In a publicly available re-analysis of the above data set, only miR-223 was confirmed to be down-regulated in macrophages, while the other miRNAs were either highly expressed or remained unchanged in macrophages, relative to monocytes (27). The differences in results from the above studies indicate that more sophisticated experimental approaches are required in the future to confidently make valid conclusions regarding these processes. Furthermore, another study also identified miR-196b and miR-1290, which targets the 3′ UTR of HIV-1 to regulate HIV-1 latency **(** **Figure 1** **)** (16). These studies suggest that a large variety of miRNAs may be responsible for regulating latency in HIV-1-infected cells.

**Table 1:** miRNAs regulating HIV-1 directly.

miRNA	Target	Action	Experimental strategy	Reference
miR-28	3′ UTR of HIV-1 mRNA	Contributes to HIV-1 latency	*In vitro*	(23)
miR-125b	3′ UTR of HIV-1 mRNA	Contributes to HIV-1 latency	*In vitro*	(23)
miR-150	3′ UTR of HIV-1 mRNA	Contributes to HIV-1 latency	*In vitro*	(23)
miR-223	3′ UTR of HIV-1 mRNA	Contributes to HIV-1 latency	*In vitro*	(23)
miR-382	3′ UTR of HIV-1 mRNA	Contributes to HIV-1 latency	*In vitro*	(23)
miR-29a	Nef	Suppresses viral replication	*In vitro*	(24, 25)

Nef is expressed early during the HIV-1 life cycle, and is indispensable for the progression of HIV-1 infection and for the release of infectious virions (28, 29). Bioinformatics tools have identified miR-29a and miR-29b to have binding sites in the Nef transcript (**Table 1**) and inhibition of miR-29a increases the replication of HIV-1 (24), indicating that miR-29a plays a role in inhibiting HIV-1 replication.

### 2.2 Host miRNAs that indirectly target HIV-1

Some miRNAs are well known to regulate host proteins that contribute to the HIV-1 life cycle, e.g., those miRNAs indirectly targeting HIV-1 infection by regulating host proteins.

#### 2.2.1 miR-146a targets CXCR4 to prevent HIV-1 entry

CXCR4 is a co-receptor of the CD4 molecule, which aids HIV-1 entry into myeloid cells and T-lymphocytes. MiR-146a directly targets CXCR4 while Promyelocytic leukemia zinc finger (PLZF) is responsible for down-regulating the expression of miR-146a **(** **Figure 1** **)** and therefore, silencing PLZF will lead to up-regulation of miR-146a. Consequently, levels of CXCR4 are decreased, which prevents HIV-1 infection of the leukemic monocyte cell line and CD4^+^ T- lymphocytes (**Table 2**) (36). Thus, a novel therapeutic strategy could potentially be devised by utilizing the preceding mechanism to prevent HIV-1 entry into cells *via* exploitation of the PLZF/miR-146a axis.

**Table 2:** miRNAs modulating HIV-1 infection by regulating host proteins.

miRNA	Target (s)	Action	Experimental strategy	Reference
miR-198	cycT1	Suppresses viral replication	*In vitro*	(30)
miR-27b	cycT1	Suppresses viral replication	*In vitro*	(31)
miR-17/92 cluster	PCAF	Suppresses viral replication	*In vitro*	(32)
miR-15a/b, miR-16, miR-20a, miR-106b, and miR-93	Pur-α	Suppresses HIV-1 infection	*In vitro*	(33)
miR-34a and miR-217	SIRT1	Increases HIV-1 infection by mediating Tat	*In vitro*	(34, 35)
miR-139-5p	FOXO1	Promotes HIV-1 infection	*In vitro*/*in vivo*	(22)
miR-155	ADAM10, LEDGF and NUP153	Reduces viral integration into host DNA	*In vitro*	(15)
miR-146a	CXCR4	Inhibits HIV entry	*In vitro*	(36)
miR-34a	PNUTS	Facilitates HIV-1 replication	*In vitro*	(37)
miR-155 and miR-181a	SAMHD1	Reactivates HIV replication	*In vitro*	(38)
miR-1236	VprBP	Regulates monocyte/DC susceptibility to HIV-1	*In vitro*	(39)
Let7c, miR-34a, miR-124a	P21, TASK1	Decreases HIV-1 infection	*In vitro*	(40)
miR-146a	CCL5	Reduces monocyte migration	*In vitro*	(21)
miR-221 and miR-222	CD4	Inhibits HIV-1 entry	*In vitro*	(14)
miR-34c-5p	Several genes	Promotes HIV-1 replication	*In vitro*	(20)

#### 2.2.2 miR-221 and miR-222 targets CD4 to restrict HIV-1 entry into macrophages

CD4 receptors that are required for viral entry are expressed on macrophages (41). However, CD4 expression by macrophages is 20-fold less, compared to CD4^+^ T-cells. RNA-seq data showed that two miRNAs, miR-221 and miR-222, are up-regulated in bystander macrophages and were found to be negative regulators of CD4 and CD4-mediated HIV-1 entry **(** **Figure 1** **)** (**Table 2**). TNF-α, which is an inhibitor of CD4, enhances the expression of both miRNAs. Inhibition of miR-221/222 enhances CD4 expression, and allows HIV-1 entry into TNF-α-treated macrophages. Therefore, both miR-221 and miR-222 restricts HIV-1 entry, and act as effectors of the antiviral host response activated during infection of macrophages (14).

#### 2.2.3 miR-155 targets ADAM10, LEDGF, and NUP153 to suppress HIV-1 genome integration into host DNA

HIV-1 relies on host dependency factors (HDFs) to facilitate infection. miR-155 targets HIV-1 HDFs before its genome is integrated into the host genome (15). miR-155 expression levels are increased when primary human macrophages are induced by TL3 or TLR4, and this subsequently results in decreased HIV-1 infectivity. miR-155 targets three HDFs i.e., ADAM metallopeptidase domain 10 (ADAM10), transcriptional co-activator lens epithelium-derived growth factor (LEDGF)/p75, and nucleoporin 153 (NUP153) in primary macrophages at their 3′ UTR (**Table 2**). Since all these three HDFs are involved in the pre-integration process of viral genome with host DNA and miR-155 targets HDFs, increased expression of miR-155 will result in anti-HIV-1 effects **(** **Figure 1** **)** (15).

#### 2.2.4 miR-15a/b, 16, 20a, miR-93, and 106b down-regulate Pur-α to suppress HIV-1 infection

Purine-rich element binding protein α (Pur-α) is an RNA and DNA binding protein which binds to Tat and TAR elements to augment HIV-1 transcription. Levels of Pur-α are lower in monocytes compared to monocyte-derived dendritic cells (DCs) because of high expression levels of miR-15a, miR-15b, miR-16, miR-20a, miR-93, and miR-106b in monocytes, which target the 3’ UTR of Pur-α (**Table 2**). Over-expression of these miRNAs significantly suppresses HIV-1 infection of monocyte-derived DCs by down-regulating Pur-α, while inhibition of these miRNAs increases Pur-α levels and removes the restriction of HIV-1 infection in monocytes **(** **Figure 1** **)** (33). Therefore, host miRNAs regulate differentiation dependent susceptibility of host cells towards HIV-1 infection.

#### 2.2.5 Downregulation of miR-17/92 cluster increases HIV-1 replication

HIV-1 Tat is required for the initiation of HIV genome transcription, and is one of the first HIV proteins that are expressed after infection starts. Tat regulatory protein requires two domains for its activity, i.e., the activation domain (AD) and the arginine rich motif (ARM). The AD is regulated by the acetylation of p300/CBP-associated factor (PCAF) at Lys-28 (42), which is an important co-factor of Tat in HIV-1 replication and is targeted by the polycistronic miRNA cluster-miR-17/92. When the miR-17/92 cluster is knocked down, HIV-1 production is increased in the Jurkat cell line (**Table 2**). More specifically, it is miR-17-5p and miR-20a of the miR-17/92 cluster that down-regulates PCAF protein and HIV-1 production **(** **Figure 1** **)**. Transfection of PBMCs with miR-11-5p and miR-20a significantly reduced both PCAF expression and HIV-1 production, and transfection of anti-miR-17-5p and anti-miR-20a increased both PCAF expression and HIV-1 production (32, 42).

#### 2.2.6 miR-27b and miR-198 target cyclin T1 to repress HIV-1 replication

The positive transcription elongation factor B (P-TEFb) is known to play a role in viral transcription by interacting with HIV-1’s Tat protein and TAR element, and is composed of two subunits, viz., cyclin dependent kinase 9 (CDK9) and cyclin T1 (cycT1). P-TEFb expression is decreased by the degradation of cycT1 or CDK9, and as a result, HIV-1 replication is inhibited in HeLa cells (43). miR-27b has been identified to target cycT1 in resting CD4^+^ T-cells (**Table 2**). The activated CD4^+^ T-cells result in down-regulation of miR-27b and a subsequent up-regulation of cycT1 **(** **Figure 1** **)**. Over expression of this miRNA results in reduced HIV-1 reporter virus viral gene expression levels and replication of the NL4.3 strain by down-regulation of cycT1 (31). Subsequently, in another study, miR-198 was identified to target cycT1 and suppress its protein levels (**Table 2**). Additionally, over expression of miR-198 decreases the replication of HIV-1 in monocytes by repressing cycT1 expression **(** **Figure 1** **)** (30).

#### 2.2.7 miR-34a and miR-217 target SIRT1 to enhance HIV-1 infection

miR-34a and miR-217 target sirtuin 1 (SIRT1) to enhance HIV-1 infection (34, 35). The Tat protein is activated when it is acetylated; however, SIRT1 mediates the regulation of Tat transactivation and HIV-1 transcription by deacetylating Tat. Tat expression leads to up-regulation of miR-34a and miR-217, where they target the 3′ UTR of SIRT1 mRNA to inhibit its expression (**Table 2**) (34, 35). Thus, inhibition of SIRT1 due to miR-34a and miR-217 will ease the restriction in terms of deacetylation on Tat, and in turn will enhance HIV-1 infection **(** **Figure 1** **)**.

#### 2.2.8 miR-34a targets PNUTS to enhance HIV-1 replication

miRNA microarray studies suggest that miR-34a is up-regulated in response to HIV-1 infection in T-cells. Furthermore, miR-34a modulates HIV-1 replication in T-cells by regulating a host protein, i.e., phosphatase 1 nuclear targeting subunit (PNUTS) (**Table 2**). miR-34a therefore can be seen to target PNUTS to promote viral replication in cells **(** **Figure 1** **)** (37).

#### 2.2.9 let7c targets p21, and miR-34a and miR-124a target TASK1 to promote viral spread

The innate host immune response delays the spread of HIV. The innate inhibitory mechanism includes several host proteins which play roles in HIV-1 infection inhibition (40). miRNA expression profiling has been performed in HIV-1-infected T-cell lines, which show that let‐7c, miR‐34a, and miR‐124a are up-regulated. The let-7c targets and down-regulates p21 (CDKN1), while miR-34a and miR-124a target and down-regulate TASK1 (KCNK3), which is among several proteins that restrict viral spread (**Table 2**). HIV-1 infection up-regulates the expression of these miRNAs, which decreases the expression levels of p21 and TASK1, and as a result, virion release and copy numbers are increased. By inhibiting these miRNAs, the levels of p21 and TASK1 are increased, resulting in decreased viral replication and spread. HIV-1 becomes more infectious by inducing miRNAs that target and down-regulate host innate immune system proteins (40).

#### 2.2.10 miR-34c-5p promotes HIV-1 replication by targeting several genes

Specific T-cell receptors (TCR) on CD4+ T-cells prime different gene expression profiles responsible for either cell activation, proliferation, and differentiation into effectors and regulatory cells, which are under the control of miRNAs (44, 45). Next generation sequencing has shown miR-34c-5p to be up-regulated in naïve CD4+ T-cells in response to TCR stimulation, which subsequently targets several genes involved in TCR signaling and cell activation **(** **Figure 1** **)** (**Table 2**). Moreover, both HIV-1 and HIV-2 have the capacity to reduce the induction of this miRNA, which was also found to be responsible for activation of cells and HIV-1 replication. These findings suggest that the down-regulation of miR-34c-5p during HIV infection delineates its anti-viral host response (20).

#### 2.2.11 miR-139-5p targets FOXO1 to promote HIV-1 infection

Extracellular vesicles (EVs) contain extra-chromosomal DNA, RNA, and various proteins (46). miR-139-5p was found to be significantly up-regulated in plasma (p) EVs from HIV-1 patients on ART, HIV-1 patients who were ART-naïve, and even in healthy controls (HCs) (22). FOXO1 inhibition promotes HIV-1 infection in resting T-cells (47). FOXO1 was observed to be down-regulated in plasma extracellular vesicles (pEVs) in treated J-Lat 10.6 cells obtained from HIV ART-naïve patients and from HIV patients on ART, but not in HCs. This indicates that pEV-associated activation of virus, especially by ART-naïve pEVs and HIV patients on ART in J-Lat 10.6 cells, occurs with the help of the miR-139-5p/FOXO1 axis **(** **Figure 1** **)** (**Table 2**) (22).

#### 2.2.12 miR-146a decreases monocyte migration by targeting CCL5

Chemokine (C-C motif) ligand 5 (CCL5) plays an important role in HIV-1 pathogenesis (21). HIV-1-infected macrophages express higher levels of miR-146a compared to uninfected cells. CCL5 is the direct target of miR-146a and is down-regulated when mir-146a is over expressed, and vice versa. Furthermore, CCL5-induced monocyte migration is reduced by miR-146a, and thus this miRNA contributes to HIV-1 pathogenesis (**Table 2**) (21).

#### 2.2.13 miR-155 and 181a reactivates HIV-1 replication by targeting SAMHD1

Generally, astrocytes are able to restrict HIV infection, and this is regulated by sterile alpha motif and histidine/aspartic acid domain-containing protein 1 (SAMHD1). miR-155/miR-181a directly targets SAMHD1 to decrease SAMHD1 expression levels and hence reactivates viral replication in astrocytes, while inhibiting these miRNAs results in increased SAMHD1 levels, and hence restricts viral replication (**Table 2**) (38).

#### 2.2.14 miR-1236 targets VprBP to restrict HIV-1 infection in monocytes

HIV-1 replicates in monocyte-derived DCs and macrophages; however, this replication is restricted in monocytes (39, 48). HIV-1 viral protein binding protein (VprBP) expression is necessary to promote HIV-1 infection. DCs express elevated levels of VprBP to facilitate viral replication. VprBP levels are undetectable in monocytes; however, VprBP mRNA levels in monocytes are even higher than levels in DCs. This suggests post-transcriptional inhibition of VprBP in monocytes. Furthermore, the expression of miR-1236 is higher in monocytes compared to DCs, and this miRNA directly targets the 3’UTR of VprBP to inhibit its expression, and restrict HIV-1 infection in monocytes (**Table 2**). Interestingly, inhibiting miR-1236 increases the translation of VprBP and HIV-1 infection in monocytes **(** **Figure 1** **)**. Similarly, the overexpression of miR-1236 decreases the translation of VprBP and HIV-1 infection in DCs. Thus, VprBP, a host factor targeted by miR-1236, regulates monocyte/DC susceptibility to HIV-1 infection (39).

#### 2.2.15 Drugs of abuse regulate miRNA expression to influence HIV-1 infection

Various stimuli can alter miRNA expression within cells to alter HIV-1 infectivity. Drugs of abuse are co-related to the HIV-1 pandemic, and affect various aspects of HIV disease. Morphine has been found to enhance HIV replication by downregulating anti-HIV miRNAs, i.e., miR-28, mir-125b, miR-150, and miR-382 in monocytes, compared to untreated cells. Heroin addicts have lower levels of expression of these miRNAs in PBMCs, compared to healthy controls. Further, monocytes treated with morphine has been shown to impair type I interferon (IFN)-induced anti-HIV miRNA expression. These findings suggest that the use of opioids compromises intracellular innate anti-HIV mechanisms in monocytes, and therefore enhances cellular susceptibility to HIV infection. (49). Similarly, cocaine serves as a cofactor to enable HIV-1 infection to progress to acquired immunodeficiency syndrome (AIDS). Cocaine contributes to HIV-1 replication in primary CD4^+^ T-cells isolated from human PBMCs. miR-125b has been shown to be significantly down-regulated in cocaine-treated CD4^+^ T-cells. The down-regulation of this miRNA enhances HIV-1 replication, while overexpression of miR-125b results in decreased viral replication (50).

Other stimuli of anti-HIV-1 miRNAs include Toll-like receptor (TLR) ligands and cytokines. TLR3, type I interferon (IFN-I), IFN-α, and IFN-β induction have been found to be responsible for the up-regulation of miR-28, mir-125b, miR-150, and miR-382 in primary macrophages to exert their anti-HIV-1 effects (51, 52).

### 2.3 miRNAs predicted to play active roles in HIV-1 pathogenesis

Apart from the direct or indirect roles of miRNAs in HIV-1 pathogenesis, some studies describe different miRNAs being involved in the pathogenesis of different diseases, for example, in cancers, hepatitis C virus (HCV) infection, dengue virus (DENV) infection, influenza, and vesicular stomatitis virus (VSV) infection (53–56). Moreover, they target genes which have known functions in HIV-1 infection. Therefore, these miRNAs could reliably be predicted to play active roles in HIV-1 pathogenesis as well.

#### 2.3.1 miR-107 may target CCR5 to regulate HIV-1 internalization

HIV-1 entry into the host cell is the first step in causing successful infection. A host factor, chemokine receptor 5 (CCR5), plays an indispensable role in HIV internalization (57). Interestingly, a deletion of 32 base pairs (bp) in the CCR5 gene has been observed in some individuals, and these individuals produce lower levels of CCR5 on the surface of their CD4^+^ T-cells, which positively correlates with resistance to HIV internalization. Therefore, CCR5 expression is pivotal in regulating HIV replication. In another study, miR-107 was found to directly target the CCR5 gene by binding to its 3′ UTR (**Table 3**) (65). It is, therefore, reasonable to assume that miR-107 expression is responsible for the regulation of HIV internalization *via* modulation of CCR5 levels.

**Table 3:** miRNAs in other diseases regulating the genes associated with HIV-1 infection.

miRNAs involved in diseases		Genes regulating HIV infection		Reference
miRNA	Relationship in disease	Gene	Effect	
miR-296-3p	Prostate cancer	ICAM 1	HIV infection	(58–60)
miR-24-3p	Colon cancer	TRIM11	Anti-HIV-activity	(56, 61)
miR-215	HCV	TRIM22	Anti-HIV-activity	(54, 62)
miR-491	Glioblastoma multiforme	TRIM28	Anti-HIV-activity	(63, 64)
miR-107	Cancer	CCR5	HIV internalization	(57, 65)
miR-132	HIV replication	MeCP2	HIV replication	(66, 67)
miR-144	Influenza virus, EMCV, VSV	TRAF6	Part of innate immune response	(53, 68)
miR-146a	DENV	TRAF6	Part of innate immune response	(55, 68)
miR-541	Abnormal proliferation and invasion of VSMCs	IRF7	Promote HIV-1 replication	(68, 69)
miR-200a/miR-200b/miR-429	Inhibit HIV-1 production	RSAD2	Promote podocyte differentiation	(70, 71)
miR-138	OSCC	ISG15	Inhibit HIV-1 replication	(72, 73)
miR-26a	Arthritis in rats	TLR3	Reduces HIV-1 infection	(15, 74)
miR-381-3p	Breast cancer	SETDB1	Inhibits HIV-1 replication	(75, 76)
miR-134-3p	OCSCs	Rab27a	Promote HIV-1 assembly	(77, 78)

#### 2.3.2 miR-132 may suppress MeCP2 to increase HIV-1 replication

HIV-1 hijacks host miRNAs to enhance infection. miR-132 is the first miRNA known to enhance HIV-1 replication (66). This miRNA is highly up-regulated in activated CD4^+^ T-cells. Upon over expression of miR-132, methyl-CpG binding protein 2 (MeCP2), the known target of miR-132, is down-regulated in Jurkat cells (**Table 3**) (66). The role of MeCP2 in the regulation of HIV-1 replication has been demonstrated previously, where its inhibition enhances HIV-1 replication (67). Therefore, the possible link is that miR-132 up-regulation suppresses MeCP2 levels, and thereby increases HIV-1 replication.

#### 2.3.3 miR-144 and miR-146a may target TRAF6 to increase HIV-1 replication

A component of the IFN signaling pathway, tumor necrosis factor receptor-associated factor 6 (TRAF6) expression is decreased in primary human macrophages after HIV-1 infection. Inhibition of expression of TRAF6 in macrophages increases HIV-1 replication, indicating that TRAF6 regulates HIV-1 production (68). In another two independent studies, miR-144 was found to decrease the replication of influenza virus, encephalomyocarditis virus (EMCV), and VSV, while miR-146a was found to decrease the replication of DENV (**Table 3**) (53, 55). Both these miRNAs directly target TRAF6, and therefore alter the antiviral landscape in cells and contribute to viral replication (**Table 3**). The assumptive deduction from the findings of these studies is that miRNA-144 and miR-146a may target TRAF6 and induce the replication of HIV-1 as well.

#### 2.3.4 miR-24-3p, miR-215, and miR-491 may target the TRIM family of proteins to regulate HIV-1 infection

The members of the family of E3 ubiquitin ligases, the tripartite motif (TRIM) proteins i.e. TRIM11, TRIM22, and TRIM28, have all been shown to have anti-HIV activity (61–63, 79). miR-24-3p suppresses the expression of TRIM11 in colon cancer, and therefore inhibits apoptosis to promote cellular proliferation (**Table 3**) (56). In HIV infection, TRIM22, through ubiquitylation, inhibits the processing of viral particles and budding. TRIM22 also has anti-HCV activity, which is regulated by miR-215 in con1b and Huh7.5.1 cells (**Table 3**) (54). Similarly, miR-491 targets TRIM28 in the pathogenesis of glioblastoma multiforme (GBM) (**Table 3**) (64). Therefore, determining the fundamental roles of miR-24-3p, miR-215, and miR- 491 with respect to HIV-related disease is likely to be a rewarding area of investigation for future study.

#### 2.3.5 miR-396-3p may target ICAM1 to regulate HIV-1 infection

Intracellular adhesion molecule 1 (ICAM1), a host factor, augments viral infectivity by directly inserting into mature HIV virions (58, 59). It has also been shown that miR-296-3p inhibits the expression of ICAM1 by directly binding to it in the malignant M12 cell line and prostate cancer cells (**Table 3**) (60). It would, therefore, be an interesting exercise to experimentally attempt to determine whether miR-296-3p reduces HIV-1 infectivity.

The predictions made from a few other examples include the over expression of interferon regulatory factor 7 (IRF7) resulting in increased HIV-1 replication in macrophages while inhibiting IRF7, which results in reduced viral replication (68). In another study, miR-541 was shown to promote abnormal proliferation and invasion of vascular smooth muscle cells (VSMCs). miR-541 targets IRF7, and down-regulation of IRF7 increases VSMC proliferation (**Table 3**) (69). In one study, Viperin (RSAD2) was observed to inhibit HIV-1 production (**Table 3**) (71), while in another study it was found that miR-200a, miR-200b, and miR-429 promote podocyte differentiation by directly targeting RSAD2, and represses its expression (**Table 3**) (70). Interferon stimulated gene 15 (ISG15) inhibits HIV-1 replication (**Table 3**) (72), while miR-138 inhibits the expression of ISG15 in oral squamous cell carcinoma (OSCC) cells (**Table 3**) (73). TLR3 reduces HIV-1 infection and miR-26a ameliorates Pristane-induced arthritis in rats by targeting TLR3 (**Table 3**) (15, 74). SET domain, bifurcated 1 (SETDB1) restricts HIV-1 replication, while another study showed that miR-381-3p suppresses SETDB1 to inhibit breast cancer progression (**Table 3**) (75, 76). Rab27a was shown to promote HIV-1 assembly by regulating plasma membrane levels of phosphatidylinositol 4,5-bisphosphate, while another study showed miR-134-3p to inhibit proliferation and cell cycle progression in human OCSCs by inhibiting the expression of Rab27a (**Table 3**) (77, 78). Therefore, the miRNAs identified in these studies targeting mRNAs that have effects on HIV infection, seem quite likely to play roles in HIV pathogenesis as well, and warrant investigative exploration in the future.

### 2.4 miRNAs associated with immune reconstitution in HIV-infected patients

Approximately 10-40% of HIV-infected patients fail to achieve meaningful or sustained improvement in immune function after ART. Therefore, investigation of potential roles that miRNAs may play in immune recovery during or after antiretroviral therapy (ART) in HIV-1 patients and immunological non-responders (INRs) has already been initiated (17, 19, 80). miRNA sponges are RNA transcripts which include long non coding RNAs (lncRNAs) and circular RNAs (circRNAs), that associate with and sequester miRNAs, thus not allowing them to interact with their target mRNAs (81). circRNAs contain several miRNA binding sites, and inhibit miRNA function by acting as miRNA sponges. In an attempt to determine the existence of biological crosstalk in competing endogenous RNA (ceRNA) networks, RNA sequencing (RNA-seq) and miRNA sequencing (miR-seq) have been performed on PBMCs from HAART-naïve patients, early HIV-infected (EHI) patients, and 3 healthy controls (HCs) (80). miR-seq analysis showed that 30 miRNAs are differentially expressed (DE) between the two groups, and among them 12 miRNAs were up-regulated and 18 miRNAs were down-regulated. Moreover, in the same study, ceRNA networks were constructed which showed that 21 DE miRNAs share miRNA response elements (MREs) with 516 DE circRNAs and 903 DE mRNAs. Furthermore, the RNA-seq data indicates that the mRNAs, CCNK, CDKN1A, and IL-15 are DE, and interestingly, these three mRNAs have already been found to play roles in HIV-1 replication. Among the DE miRNAs, miR-27b-3p, miR-542-3p, miR-101-3p, let-7c-5p, and miR-548ah-3p have been predicted to have MRE binding sites on the preceding mRNAs. Similarly, 67 DE circRNAs were predicted to have shared MREs with CCNK, CDKN1A, and IL-15 mRNAs. Among these 67 circRNAs, 18 were predicted to share MREs of miR-27b-3p with CCNK, 40 were predicted to share MREs of let-7c-5p, miR-101-3p, and miR-542-3p with CDKN1A, and 9 were predicted to share MREs of miR-548ah-3p targeting IL-15. Therefore, HIV replication seems to be regulated by miRNA sponging activity in PBMCs in HAART-naïve patients.

In another study, the association between miRNAs and immunological nonresponse was assessed by investigating the miRNA profiles in immunologically reconstituted HIV-1 patients and immunological HIV-1 non-responders (17). miRNAs were analyzed using next-generation sequencing, and differences in expression profiles at baseline and after 24 months of maintaining virological suppression (VS) were measured between immunological responders (IR) and INRs. It was observed that, compared to IRs, a substantial proportion of miRNAs were downregulated in INRs. The miRNA, let-7d-5p, was downregulated in 9 INRs (total INRs=11), but only in 2 IRs (total IRs=13). There is therefore the potential for the miRNA let-7d-5p to be used as a potential biomarker for INRs (17).

One study attempted to investigate miRNAs as predictive markers that may be useful in identifying patients with poor immune response earlier (19). Plasma samples were taken before ART initiation for miRNA isolation and sequencing. It was observed that miR-580, miR-627, miR-138-5p, miR-16-5p, and miR-323-3p are upregulated in INRs, in contrast to observations in IRs. The expression levels of miRNAs negatively correlate with both CD4^+^ T-cell counts and increased proportions of CD4^+^ T cells after one year of ART, and could potentially be used to differentiate IRs from INRs. Therefore, miRNAs could possibly be used to accurately predict immune response after ART (19).

## 3 Controversy surrounding expression of miRNA by HIV-1

Several viruses encode viral miRNAs (vmiRNAs) which play roles in viral replication, host immune evasion, and survival within a host (82, 83). However, whether RNA viruses actually encode miRNAs at all is debatable (84, 85). One of the reasons for this controversy is because RNA-induced silencing complex- (RISC)-mediated degradation of the non-coding region responsible for a functional miRNA will result in the degradation of the entire viral RNA, and therefore there is an absence of a non-coding region (86). Another reason could be that RNA viruses replicate within the cytoplasm of cells, and are therefore not processed by the Drosha enzyme and transactivation response element RNA binding protein (TRBP). Nevertheless, HIV-1, being a retrovirus, can be reverse transcribed into double stranded DNA and imported into the nucleus where it integrates into the host genome. Therefore, HIV-1 has access to the RNA interference (RNAi) machinery of both the nucleus and the cytoplasm, similar to host miRNAs.

Bioinformatics tools have suggested that HIV-1 encodes five pre-miRNA candidates (87). Moreover, Nef, the viral protein, is also supposed to encode a vmiRNA (Nef-U3-miR-N367) that degrades Nef and decreases viral replication (88, 89). Another vmiRNA, hiv1-miR-H1, encoded in the 3′-end of the HIV-1 genome, has been reported to target cellular miR-149 that can target Vpr of HIV-1 (90). Similarly, the protein coding regions of Gag-Pol and Env proteins of HIV-1 can encode several miRNA-like sequences (91). In HIV-infected T-lymphocytes, viral RNA accumulation has been analyzed *via* a sensitive SOLID™ 3Plus System, and several small non-coding RNAs (sncRNAs) derived from the genome of HIV-1 have been detected. These sncRNAs were either vmiRNAs or viral short interference RNAs (vsiRNAs) (92). In another study, a sequence-targeted enrichment strategy was used to identify sncRNAs derived from HIV-1 infected primary CD4^+^ T-lymphocytes and macrophages and hundreds of sncRNAs from 16 to 89 nucleotides in length were detected (93). Moreover, the transactivation RNA (TAR) element, which recruits Tat in infected cells, has been determined to interact with Dicer, and is processed by Dicer, and generates vmiRNAs that regulate chromatin remodeling of viral LTR (87, 94). Two other vmiRNAs, vmiR88 and vmiR99, are encoded by HIV-1 LTR through a non-canonical miRNA pathway, and are able to trigger tumor necrosis factor alpha (TNF-α) release by macrophages (95).

Evidence endorsing the fact that HIV-1 does not encode short interference RNA (siRNAs) or miRNAs in infected T-cells, primary peripheral blood mononuclear cells (PBMCs), and macrophages is provided in other studies (96, 97). Advanced and improved detection methods are therefore indispensable to identify HIV-1 encoded miRNAs.

## 4 Therapeutic potential of miRNAs for HIV-1

Different phases of the HIV viral life cycle have been targeted in developing the multiple antiretroviral drugs utilized in modern ART. ART has been successful in controlling viral replication in the majority of HIV-infected patients (98). Extensive progress has been made with ART; however, infection by HIV-1 remains a global menace, and has the real and unavoidable risk of being incurable in most patients. Besides, all ART drugs have adverse effects and the potential for drug toxicity, and the emergence of HIV drug resistance poses a substantial threat to the overall success of current ART regimens (99–101). These concerns have compelled investigators to attempt to identify novel therapeutics in the quest to cure HIV-1.

RNA-based therapeutics have garnered much attention in recent times because of its reported success in treating several types of diseases. Potential miRNA drugs, with reported success in treating many types of infectious and non-infectious diseases, have been reported in the literature (102–106). Recent studies have shown that dysregulation in miRNA profiles affect HIV replication (17, 62, 71, 72, 107, 108). Despite being aware of the diverse roles of the many miRNAs intricately woven into the fabric of the HIV life cycle, the practical application of miRNAs as interventional therapeutics and diagnostics remains a largely unexplored and untapped area of HIV/AIDS research. The use of miRNAs as a treatment strategy is an emerging field of research endeavor, and very few miRNA-associated drugs have thus far been approved for clinical applications by the United States Food and Drug Administration (FDA) (109). For future study, it would be of considerable interest to explore the therapeutic potential of miR-34a, as this particular miRNA targets three host proteins (PNUTS, SIRT1, and TASK1) to promote HIV-1 infection (**Figure 1**).

## 5 Conclusions

To sum up, numerous miRNAs target HIV-1 either directly or indirectly and play significant roles in the pathogenesis of HIV infection and progression. These miRNAs are involved in different processes of HIV-1 infection and effect different impacts on the pathogenesis of HIV-1 infection. For example, miR-146a, miR-221 and miR-222 down regulate host factors to prevent HIV-1 entry into host cells; miR-155 down regulates host proteins to inhibit HIV-1 genome from integrating into host genome; miR-34a, miR-139-5p, miR-155, mir-181a and miR-217 suppress different host proteins to increase HIV-1 replication; and miR-17/92 cluster, miR-27b and miR-198 down regulate host proteins to decrease HIV-1 replication; miR-34a targets three host proteins to promote HIV-1 pathogenesis, whereas miR-24-3p, miR-107, miR-132, miR-144, miR-146a, miR-215, miR-396-3p and miR-491 may play predictive roles in HIV-1 pathogenesis by targeting genes that are favorable to HIV-1 infection. Furthermore, some miRNAs, such as let-7d-5p, are associated with immune reconstitution in HIV-infected patients and could be used as a potential biomarker for INRs. However, much remains unclear and warrants further study regarding the involvement of miRNAs in HIV-1 pathogenesis. Firstly, although it is already known that cell-type-specific expression of miRNAs and HDFs contribute to HIV-1 susceptibility, it is not known whether cell differentiation and cell activation status are involved in the process. Secondly, even though it is known that numerous miRNAs play roles in HIV-1 life cycle and pathogenesis, the potency and expression levels of miRNAs remain largely unknown. Thirdly, it is known that some miRNAs contribute to the establishment of HIV-1 latency, however, the degree of participation of other factors such as transcriptional inefficiencies have not been determined. Fourthly, researchers found that miR-27b repressed HIV-1 replication by down regulating cyclin T1, however, the role of miR-27a, which differed from miR-27b only by one nucleotide at the 3′ end, was undermined (31). And fifthly, in the studies describing the association of miRNAs and immune reconstitution in HIV-infected patients (17, 80), the sample sizes were small and specific cells types were not used for analysis, indicating that the study conclusions warrant further investigation.

## 6 Future perspectives

In the future, the potential application of miRNAs may be of great interest to investigators, and miRNA mimics and inhibitors which specifically target their respective mRNAs could be used to inhibit HIV-1 replication, infectivity and pathogenicity, or to boost immune reconstitution of people living with HIV. In addition, differentially expressed miRNAs during HIV-1 infection could also be used to guide researchers to explore host proteins that may be manipulated for therapeutic benefits. However, some issues need to be fully addressed in order to achieve these goals. Firstly, one miRNA may target several host genes. For example, miR-34a can target three different host genes, i.e. SIRT1, PNUTS and TASK1, in its regulation of HIV-1 infection (35, 37, 40), and therefore, it is reasonable to speculate that miR-34a mimics may result in off-target effects when used *in vivo*, which may pose a challenging obstacle for its potential future clinical application. Secondly, a potential Achilles’ heel of a potential miRNA-based HIV therapy is related to the lack of a targeted delivery system. miRNAs, by nature, are inherently structurally unstable, and therefore, it is unimaginable for them to be able to strike their targets with a high degree of accuracy without an efficient, targeted delivery system that can substantially enhance their half-life and can accurately guide them to their targets. And finally, another compelling research area in the future that warrants future investigation is that miRNAs may be used as biomarkers or predictive markers of immune response after ART. We believe that as research deepens in the future, the therapeutic application of miRNAs will eventually come true.

## Author contributions

FR and YC conceptualize the study. FR wrote the manuscript. SZ revised the manuscript and figure. FS and YC revised the manuscript and approved the final draft. All authors contributed to the article and approved the submitted version.
